# Germline Deletion of Pantothenate Kinases 1 and 2 Reveals the Key Roles for CoA in Postnatal Metabolism

**DOI:** 10.1371/journal.pone.0040871

**Published:** 2012-07-17

**Authors:** Matthew Garcia, Roberta Leonardi, Yong-Mei Zhang, Jerold E. Rehg, Suzanne Jackowski

**Affiliations:** 1 Department of Infectious Diseases, St. Jude Children’s Research Hospital, Memphis, Tennessee, United States of America; 2 Department of Biochemistry and Molecular Biology, Medical University of South Carolina, Charleston, South Carolina, United States of America; 3 Department of Pathology, St. Jude Children’s Research Hospital, Memphis, Tennessee, United States of America; University of Tokyo, Japan

## Abstract

Pantothenate kinase (PanK) phosphorylates pantothenic acid (vitamin B_5_) and controls the overall rate of coenzyme A (CoA) biosynthesis. *Pank1* gene deletion in mice results in a metabolic phenotype where fatty acid oxidation and gluconeogenesis are impaired in the fasted state, leading to mild hypoglycemia. Inactivating mutations in the human *PANK2* gene lead to childhood neurodegeneration, but *Pank2* gene inactivation in mice does not elicit a phenotype indicative of the neuromuscular symptoms or brain iron accumulation that accompany the human disease. *Pank1/Pank2* double knockout (dKO) mice were derived to determine if the mild phenotypes of the single knockout mice are due to the ability of the two isoforms to compensate for each other in CoA biosynthesis. Postnatal development was severely affected in the dKO mice. The dKO pups developed progressively severe hypoglycemia and hyperketonemia by postnatal day 10 leading to death by day 17. Hyperketonemia arose from impaired whole-body ketone utilization illustrating the requirement for CoA in energy generation from ketones. dKO pups had reduced CoA and decreased fatty acid oxidation coupled with triglyceride accumulation in liver. dKO hepatocytes could not maintain the NADH levels compared to wild-type hepatocytes. These results revealed an important link between CoA and NADH levels, which was reflected by deficiencies in hepatic oleate synthesis and gluconeogenesis. The data indicate that PanK1 and PanK2 can compensate for each other to supply tissue CoA, but PanK1 is more important to CoA levels in liver whereas PanK2 contributes more to CoA synthesis in the brain.

## Introduction

Coenzyme A (CoA) is a ubiquitous cofactor that activates acyl groups and participates in several metabolic processes, including fatty acid and lipid synthesis, fatty acid degradation, ketone metabolism and the tricarboxylic acid cycle. CoA is derived from the vitamin pantothenate, and CoA synthesis is governed by the pantothenate kinases (PanKs), of which there are four active isoforms in mammals: PanK1α, PanK1β, PanK2 and PanK3 [1]. The PanK1α and β isoforms are derived from a single gene by use of alternate initiation exons [2], and PanK2 and PanK3 are encoded by separate, distinct genes [3], [4]. In contrast, most bacteria [1], fungi [5], [6], and flies [7] have one gene encoding PanK.

Information about the physiological functions of the individual mammalian PanK isoforms is just emerging. PanK1 is the most insensitive among the PanK isoforms to acetyl-CoA feedback inhibition and its expression is highest in liver and kidney. Correspondingly, CoA levels are highest in liver and kidney [8] which are the organs that provide glucose to the body during a fast. Limitation of the CoA supply by genetic deletion of PanK1 activity results in a 40% reduction in liver CoA, blunts the CoA increase in response to fasting, and impairs gluconeogenesis during a prolonged fast [9]. PanK2 is the most stringently regulated among the PanK isoforms, being the most sensitive to inhibition by acetyl-CoA [10], and its expression is highest in testes [11], thus explaining the observed defect in sperm production in PanK2-null mice [12].

Mutations in the human *PANK2* gene result in Pantothenate Kinase Associated Neurodegeneration (PKAN) disease [13], a childhood neurodegeneration that leads to parkinsonism, dystonia, dementia and ultimately death. While many of the PKAN mutations are predicted to result in truncated proteins, several point mutations either maintain or only partially reduce enzyme activity [14], while other mutations destabilize and promote PanK2 protein degradation [15], [16]. A *Pank2* knockout mouse was generated using gene disruption [12], but did not exhibit any behavioral or movement disorder, and did not accumulate iron in the brain, unlike the human PKAN disease. The human and mouse PanK2s possess similar biochemical properties, and both are inhibited by acyl-CoA and activated by acylcarnitine [10]. However, the mouse PanK2 is localized primarily in the cytosol whereas the human PanK2 is associated with mitochondria [11]. This finding raises the possibility that the cellular location of PanK2 is important for its function. On the other hand, deprivation of pantothenate elicits a movement disorder in mice [17], suggesting that overall reduction of the CoA supply may have a role in PKAN disease. Nothing is known about the impact of *Pank2* gene inactivation on CoA levels.

We generated a *Pank2* knockout mouse using *Cre-lox* methodology, which resulted in removal of exon3 that encodes a portion of the catalytic domain. We then derived a *Pank1/Pank2* double knockout (dKO) mouse in an effort to elicit a PKAN-like phenotype and address the contribution of PanK2 activity to CoA biosynthesis. Unlike the phenotypes associated with either the *Pank1* or *Pank2* single knockouts, we found that loss of both PanK1 and PanK2 activities in mice had a severe impact on postnatal physiology and development, ultimately resulting in death before weaning.

## Results

### Derivation and General Characteristics of *Pank2* Knockout Mice

**Figure 1:**
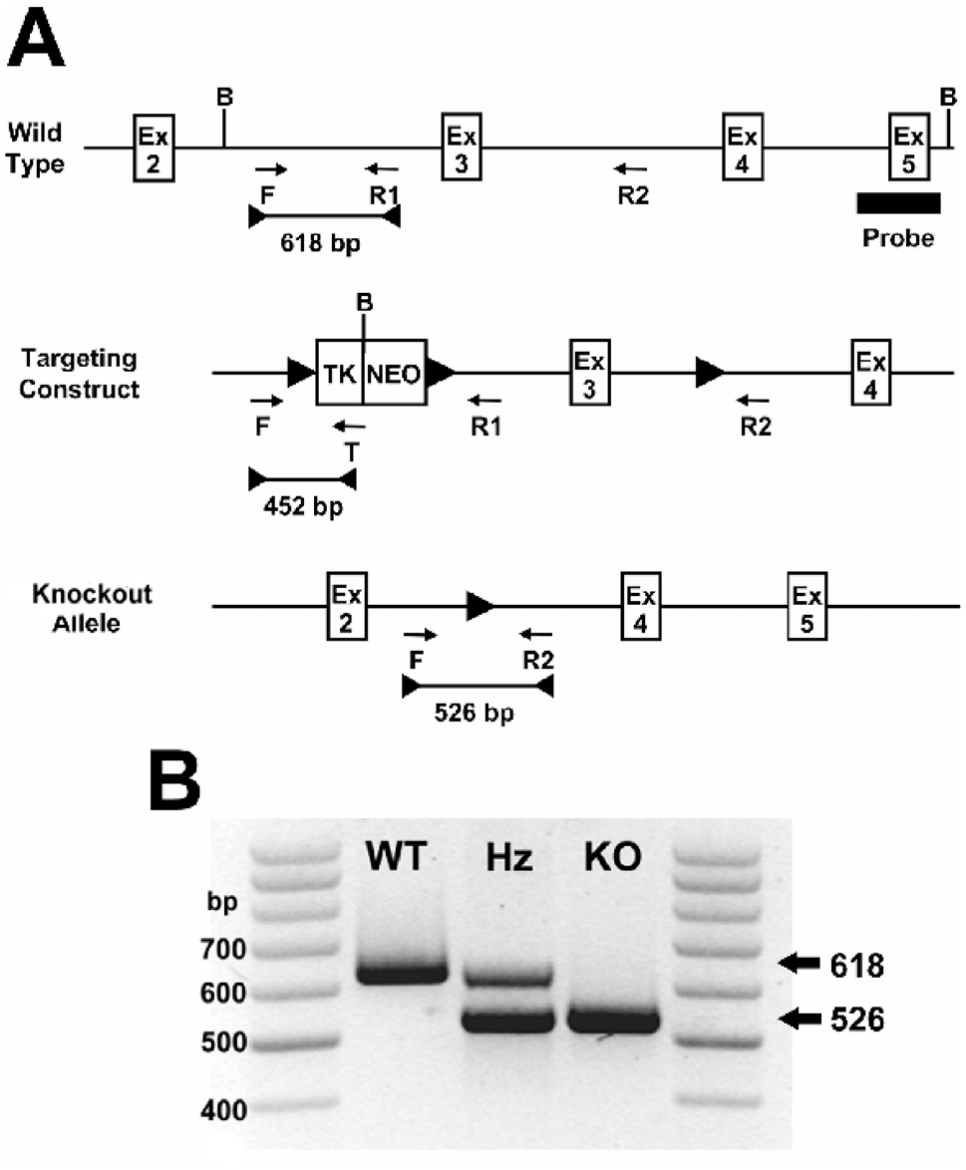
Targeting the *Pank2* gene for deletion in mice. (**A**) A selection cassette containing cDNA encoding thymidine kinase (TK) and neomycin resistance (NEO) was inserted into the intron between exons 2 (Ex2) and 3 (Ex3) of the *Pank2* gene. The cassette was flanked by *loxP* (▸) sites. A third *loxP* site was inserted between exons 3 (Ex3) and 4 (Ex4). Cre-recombinase-mediated deletion of the DNA between the outermost *loxP* sites resulted in a knockout (KO) allele which lacked Ex3 and lacked the selection cassette. Locations of the primers (arrows) for PCR genotyping are shown. PCR products and sizes are indicated (bars), and B indicates the presence of a BamHI site. (**B**) A PCR product of 618 bp using primers F and R1 indicated a wild-type (WT) allele. A PCR product of 526 bp using primers F and R2 indicated a deleted (KO) allele. Heterozygous (Hz) mice had both alleles.

The murine *Pank2* gene was modified by insertion of *loxP* sites in the introns flanking exon 3 (Fig. 1A). Recombinant embryonic stem cells from mouse strain 129 SvEv containing the modified *Pank2* gene were obtained following transfection with the floxed construct and selection in geneticin (G418), and were injected into C57BL6 blastocysts. Germline transmission of the modified *Pank2* gene was confirmed in chimeric pups. *Pank2*(flox/+) mice were bred with FVB/N-Tg(ACTB-cre)2 Mrt/J transgenic mice (Jackson Laboratories), and pups with a *Pank2* allele in which exon 3 was deleted via Cre recombinase-dependent DNA excision were obtained. The deletion resulted also in the removal of the cDNA cassette used for selection of the recombinant embryonic stem cells. *Pank2*(+/−) heterozygous mice were mated together and pups with homozygous deletion (knockout) of the Pank2 gene were identified (Fig. 1B) in the expected Mendelian ratio. The *Pank2* adult knockout mice did not exhibit gross defects in anatomy or behavior, with the exception of azoospermia as reported previously for *Pank2* knockout mice [12].

**Figure 2:**
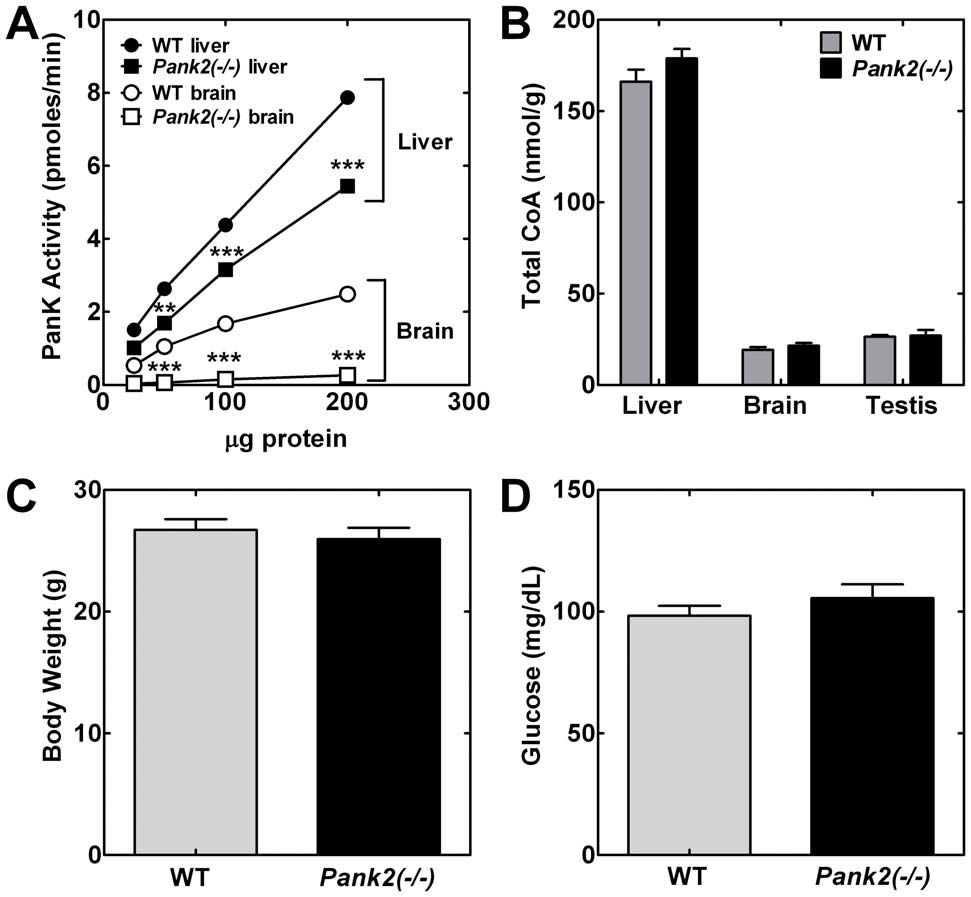
Characteristics of the *Pank2* knockout mouse. (**A**) Total PanK activity (pmoles/min) was determined in liver (•, ▪) and brain (○, □) homogenates from adult wild-type (WT,• and ○; n = 3–4) or *Pank2(−/−)* single knockout (▪ and □; n = 3–4) mice as a function of protein concentration. (**B**) Total CoA levels per gram (g) tissue from brain, liver and testes were measured in adult wild type (WT; n = 3) and *Pank2(−/−)* single knockout mice (n = 3). (**C**) Body weight (g) and (**D**) blood glucose (mg/dl) were measured in adult wild-type (WT; n = 10) and *Pank2* knockout (n = 9) adult mice. Statistically significant differences presented as * p<0.05; ** p<0.01; *** p<0.001.

Significant reduction of the total PanK enzyme specific activity in the livers (about 30%) and brains (about 90%) from the *Pank2*(−/−) animals (Fig. 2A) indicated the contribution of PanK2 activity to the total. The brain PanK specific activity was lower than that found in liver, and the data indicated that PanK2 was the major contributor to overall PanK activity in brain, whereas PanK2 constituted a smaller relative portion of the total activity in liver. After the testes, *Pank2* transcripts are highest in brain, but the CoA levels in the liver, brain and testes were not reduced in adult *Pank2* knockout mice compared to wild-type controls (Fig. 2B), indicating that the PanK1 and/or the PanK3 isoforms were sufficient to maintain the CoA levels in these tissues. Although the CoA per gram of testes was the same in wild-type and *Pank2*(−/−) mice, the sizes of the testes in the KO mice were substantially smaller and the males were sterile. We were unable to confirm histological evidence of retinal degeneration or significant body weight reduction (Fig. 2C) in the *Pank2* knockout animals up to age 6 months compared to littermate controls, in contrast with the previous report [12]. Blood glucose measurements did not differ between *Pank2*(−/−) and matched adult mice either in the fed (Fig. 2D) or fasted state (data not shown). Evaluation of the *Pank2*(−/−) adult mice did not reveal a movement disorder, and histological analyses of brain sections were unremarkable.

### Derivation and General Characteristics of *Pank1*/*Pank2* dKO Mice

**Figure 3:**
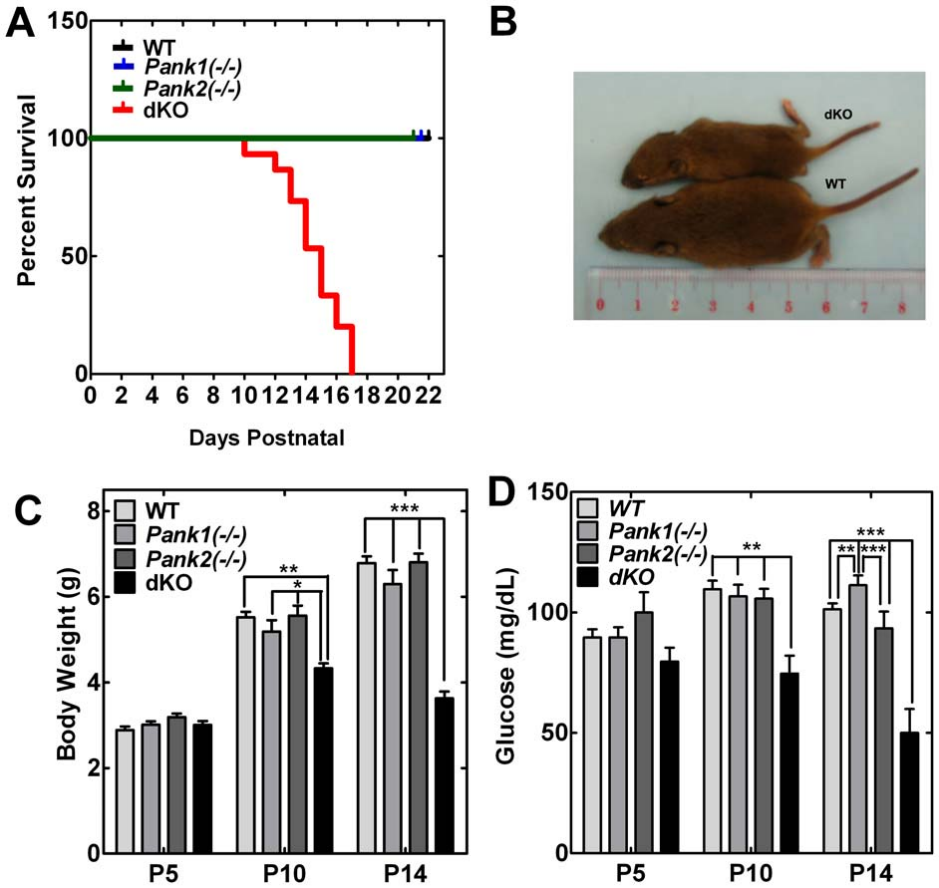
Characteristics of the dKO mice. (**A**) Postnatal survival of wild-type (black, n = 10), *Pank1(−/−)* (blue, n = 10), *Pank2(−/−)* (green, n = 5), and dKO mice (red, n = 15). (**B**) Size of a typical double knockout mouse (dKO, upper) and a wild type (WT, lower) control mouse at P10. (**C**) Body weights of wild-type (WT, n = 5, 7, 7 at P5, P10, P14, respectively), *Pank1(−/−)* (n = 7, 7, 6 at P5, P10, P14, respectively), *Pank2(−/−)* (n = 4, 4, 4 at P5, P10, P14, respectively), and dKO (n = 13, 9, 6 at P5, P10, and P14, respectively) mice. (**D**) Blood glucose measurements from wild type (n = 8, 8, 8 at P5, P10, P14, respectively), *Pank1(−/−)* (n = 5, 7, 8 at P5, P10, P14, respectively), *Pank2(−/−)* (n = 4, 8, 4 at P5, P10, P14, respectively) and dKO (n = 7, 7, 5 at P5, P10, P14, respectively) mice. Significance calculated with students t-test * p<0.05; ** p<0.01; *** p<0.001.

The *Pank1*(−/−) mice were derived and characterized recently [9]. *Pank2*(−/−) females were mated with *Pank1*(−/−) males to obtain pups heterozygous for both genes. Mating of the double heterozygous mice produced double knockout mice (dKO) with deletion of both the *Pank1* and *Pank2* genes in the expected Mendelian ratio. However, all of the dKO mice died within 18 days after birth (Fig. 3A). The dKO pups were the same weight at birth, compared to littermate *Pank1*(−/−) or *Pank2*(−/−) single knockouts and age- and sex-matched wild-type mice with the same mixed genetic background, but the dKO mice displayed a growth deficit after about 1 week, characterized by significantly reduced body size and weight (Fig. 3B and C). The dKO mice were unable to maintain serum glucose levels and became significantly hypoglycemic by 10 days (Fig. 3D). The hypoglycemia became progressively more severe and death occurred between postnatal day 10 and 17 (Fig. 3A). The viable dKO mice were observed suckling through 17 days and at the times of death, milk was present in their stomachs. Necropsy revealed mature intestinal epithelia (not shown). Rescue of the dKO pups (n = 8) was attempted by twice daily intraperitoneal injection of glucose (50 µl 5% dextrose in PBS) for two weeks starting at day 4. The dKO mice on glucose supplementation did not increase size or weight after day 7 and were hypoglycemic prior to death. The glucose supplementation was not therapeutic, similar to the results with pups deficient in mitochondrial trifunctional protein which die within 3 days after birth [18]. Despite normal organ sizes at birth, several organs in the double knockout animals were notably smaller in size by postnatal day 10, relative to whole body size, including the liver, spleen and kidney (Table 1). In contrast, the weights of the brain and heart were not significantly different between wild type and dKO animals.

**Table 1:** Organ weights in P10 wild-type and dKO mice.

	Wild Type (n = 9)		dKO (n = 8)	
Organ	Weight (mg)	Ratio^a^	Weight (mg)	Ratio
Brain	0.307±0.017	0.056±0.003	0.255±0.017	0.059±0.004
Liver	0.204±0.001	0.037±0.002	0.100±0.017	0.023±0.004*** b
Spleen	0.037±0.017	0.007±0.003	0.010±0.017	0.002±0.004*
Kidney	0.068±0.011	0.012±0.002	0.033±0.017	0.008±0.004*
Heart	0.040±0.005	0.007±0.001	0.034±0.017	0.008±0.004

a Organ weight divided by body weight.

b Significance calculated using students t-test.

* p<0.05;

*** p<0.001.

### Hepatic *Pank* Expression, PanK Activity and CoA Levels in dKO Mice

**Figure 4:**
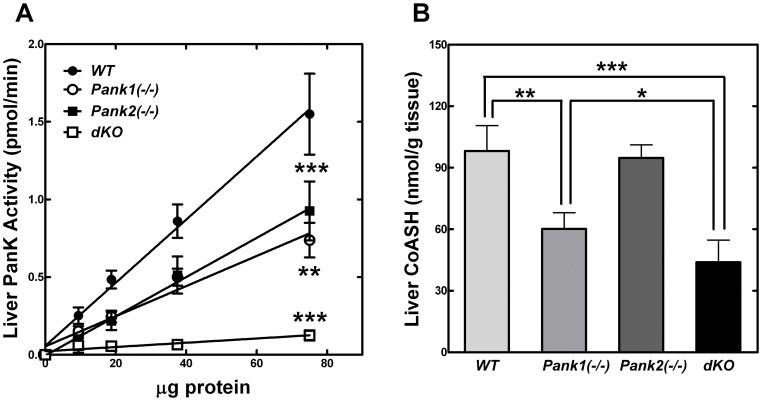
Liver PanK activity and CoA levels. (**A**) PanK enzyme activity in liver homogenates from wild-type (WT,•, n = 4), *Pank1(−/−)* (o, n = 4), *Pank2(−/−)* (▪, n = 4) and dKO mice (dKO, □, n = 4). (**B**) Liver free (unacylated) CoASH levels from wild-type (WT, n = 4), *Pank1(−/−)* (n = 4), *Pank2(−/−)* (n = 4) and dKO mice (n = 4) at age P10. * p<0.05; ** p<0.01; *** p<0.001.

Maintenance of glucose levels is a prime function of the liver. Due to the hypoglycemia which developed in the dKO pups, we examined the factors that regulate CoA levels in liver. Previously, adult *Pank1*(−/−) mice were found to exhibit reduced hepatic CoA levels, a reduced rate of CoA-dependent fatty acid oxidation in the fasted state, impaired gluconeogenesis, and mild hypoglycemia in the fasted state [9]. The hypoglycemia of the dKO mice was evident by postnatal day 10 (Fig. 3D) and so we examined the factors that control the hepatic CoA levels in postnatal day 10 (P10) pups. *Pank1* and, to a lesser extent, *Pank3* are the major transcripts expressed in liver, while *Pank2* transcripts are the least abundant in adult mice [11]. Similarly, *Pank1* expression was highest among the *Pank* isoforms in P10 wild-type liver, with *Pank2* and *Pank3* transcripts expressed at 20% and 25% of the *Pank1* level, respectively (Table 2). Expression of *Pank3* transcripts did not change in the dKO livers, but expression of the nudix hydrolases *Nudt7* and *Nudt19* was reduced significantly. These nudix hydrolases degrade acyl-CoAs [19], [20], [21], so their decreased expression suggested a compensatory adaptation to help maintain tissue CoA levels in the absence of PanK1 and PanK2 activities. The PanK enzyme activity in liver was measured in matched wild-type, *Pank1*(−/−) and *Pank2*(−/−) single knockouts, and *Pank1/Pank2* dKOs at P10 (Fig. 4A). The single knockouts had significantly reduced activity, at about 50% the amount measured in wild-type livers. The total PanK activity in the dKO livers was reduced even further, to about 10% compared to wild-type livers. These data indicated that the PanK1 and the PanK2 isoforms were responsible for the majority of the total PanK activity in liver.

**Table 2:** *Pank* and *Nudt* transcript expression in wild-type and dKO mouse liver.

Gene	Wild Type	dKO
*Pank1*	0.048±0.020 (7)	ND^a^ (7)
*Pank2*	0.010±0.002 (7)	ND^a^ (7)
*Pank3*	0.016±0.006 (7)	0.019±0.006 (7)
*Nudt7*	0.021±0.004 (8)	0.008±0.003(6)***
*Nudt19*	0.029±0.009 (6)	0.013±0.008 (5)*

Transcript abundance was measured relative to *Gapdh* (Glyceraldehyde 3-phosphate dehydrogenase) as a calibrator. Transcripts were measured in triplicate and combined results are shown ± s.d. (number of mice). Significance calculated using Students t-test.

* p<0.05;

*** p<0.001.

a ND not detectable.

Next, the liver CoA levels were evaluated in the four genotypes at P10 (Fig. 4B). Free CoASH (unacylated) was measured reliably, but the amounts of acyl-CoA were below the level of detection in the assay due to the limited amounts of material from the pups. CoASH constitutes the largest fraction of the total CoA pool and is the most sensitive to inhibition of CoA biosynthesis or loss of *Pank1* expression [8], [9]. Liver CoASH was reduced by about 40% in the *Pank1*(−/−) pups, whereas the *Pank2*(−/−) pups showed no significant reduction in liver CoASH compared to wild-type control animals, similar to the adult *Pank2*(−/−) mice (Fig. 2B). PanK1 is the predominant isoform expressed in liver (Table 2), and is the least sensitive to feedback inhibition by acetyl-CoA. The data suggest that PanK1 and PanK3 proteins may increase cellular activity through biochemical mechanisms to maintain liver CoA homeostasis in the absence of PanK2. The only known biochemical mechanisms that modify the activities of the PanKs involve interaction between the proteins and small molecule metabolic inibitors, e.g., acetyl-CoA, or activators, e.g., acyl-carnitine. During preparation of the liver lysates, prior to in vitro assay of PanK activity, small molecules were removed as described in Materials and Methods. On the other hand, measuring CoA levels in intact cells and tissues reflected the regulated PanK activities because the small molecule modifiers were not removed. The CoASH levels in the dKO livers were reduced even lower than the CoASH in the *Pank1*(−/−) single knockouts (Fig. 4B), but not as much as would be expected from the in vitro PanK activity measurements alone. If one relied solely on the in vitro PanK activity measurements, one would expect the CoASH levels to be about 90% lower in the dKO animals, compared to wild type animals. However, the CoASH levels were about 55% lower in the dKO animals. The reduction in the expression of the nudix hydrolases *Nudt7* and *Nudt19* (Table 2), which are CoA-degrading enzymes, helped to maintain the CoASH levels in the dKO anmals. These data support the concept that the CoA levels are a reflection not only of the PanK enzyme activities and their respective sensitivities to regulatory ligands, but also the activities of the nudix hydrolases. These data also suggest that a minimal CoA threshold level is required for maintaining serum glucose homeostasis. Whereas the dKO hepatic CoASH levels were about 50% lower compared to wild-type (Fig. 4B), the CoASH levels were reduced even more by 80–90% in wild-type animals treated with an acute inhibitor of CoA biosynthesis [8]. In both cases the animals died following severe hypoglycemia, which would put the hepatic CoASH threshold at >50% of wild-type levels.

### Brain *Pank* Expression, PanK Activity and CoA Levels

**Figure 5:**
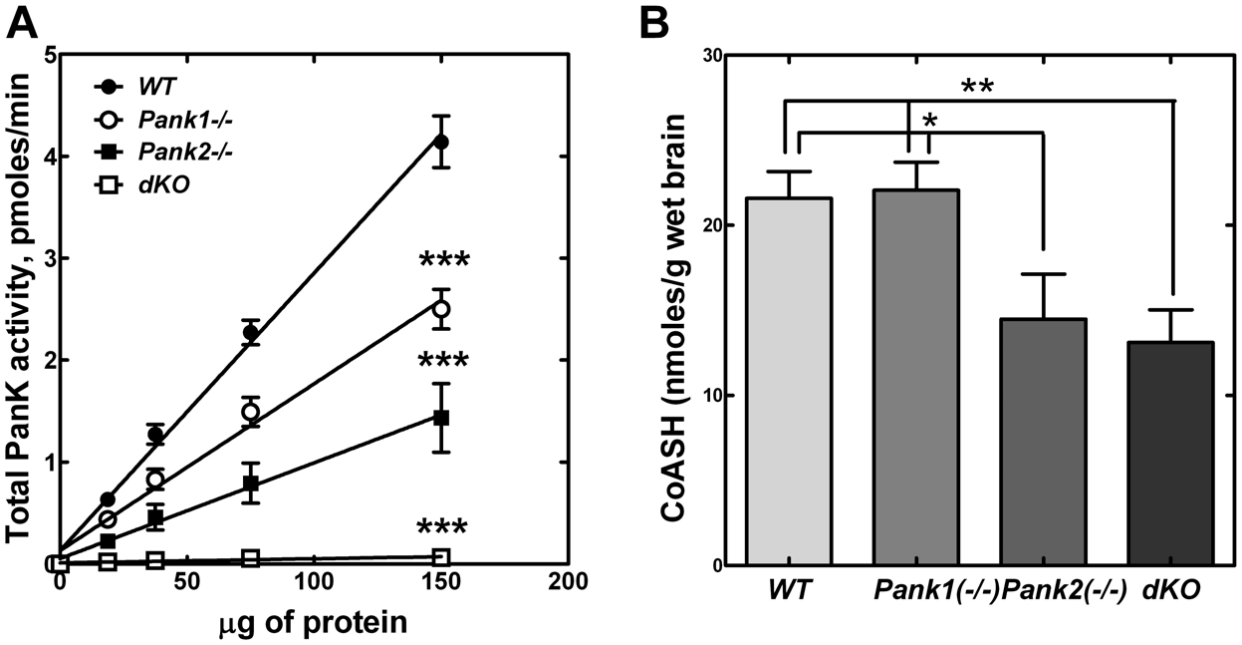
Brain PanK activity and CoA levels. (**A**) PanK enzyme activity in brain homogenates from wild type (WT,•, n = 4), *Pank1(−/−)* (○, n = 5), *Pank2(−/−)* (▪, n = 7) and dKO mice (□, n = 4). (**B**) Free (unacylated) CoASH levels from wild type (WT, n = 6), *Pank1(−/−)* (n = 6), *Pank2(−/−)* (n = 6) and dKO (n = 6) mice at P10. * p<0.05; ** p<0.01; *** p<0.001.

The factors that regulate brain CoA levels in wild-type, *Pank1*(−/−) and *Pank2*(−/−) single knockout mice, and the dKO mice were examined at P10. *Pank1* was expressed at very low levels, whereas *Pank2* and *Pank3* transcripts were predominant in wild-type brain (Table 3). The expression of *Pank3* did not change in the dKO brains. The CoA-degrading enzyme *Nudt7* was not detected in brain, and the expression of *Nudt19* transcripts was very low, but still significantly reduced in the dKO pups. As in liver, the data suggested an adaptive reduction of *Nudt19* to compensate for loss of PanK1 and PanK2 activities in the dKO brain. The total PanK activity was measured in brain homogenates (Fig. 5A) and deletion of *Pank1* resulted in about 40% reduction in activity. Deletion of *Pank2* caused about 60% decrease in PanK activity, and loss of both *Pank1* and *Pank2* resulted in >90% reduction compared to wild-type brain homogenates. The CoASH levels in *Pank2*(−/−) and dKO P10 brains were significantly reduced compared to wild-type and *Pank1*(−/−) pups (Fig. 5B). The CoA level in the dKO brains was surprising in light of the very low measurable PanK3 enzyme activity (Fig. 5A). The substantial amount of CoASH measured in the dKO brains indicated that the PanK3 isoform contributed significantly to CoA biosynthesis and the low expression of *Nudt19* (Table 3) suggested that CoA turnover was low. Although low, *Nudt19* expression was significantly reduced in the brains of the dKO animals (Table 3), which helped to maintain the CoASH levels. The amount or rate of CoA production provided by PanK3 activity was insufficient to support postnatal metabolism, however. These data indicated that PanK2 was the major isoform that contributed to brain CoA levels during the postnatal period and are in contrast with the measurements of brain CoA levels in adult *Pank2*(−/−) mice (Fig. 2B), suggesting a difference in CoA regulation between newborn and adult animals.

**Table 3:** *Pank* and *Nudt* transcript expression in wild-type and dKO mouse brain.

Gene	Wild Type	dKO
*Pank1*	0.003±0.001 (4)	ND^a^ (4)
*Pank2*	0.015±0.003 (4)	ND^a^ (4)
*Pank3*	0.024±0.004 (4)	0.026±0.003 (4)
*Nudt7*	ND a	ND (4)
*Nudt19*	0.002±0.001 (6)	0.001±0.001 (4)**

Transcript abundance was measured relative to *Gapdh* (Glyceraldehyde 3-phosphate dehydrogenase) as a calibrator. Transcripts were measured in triplicate and combined results are shown ± s.d. (number of mice). Significance calculated using Students t-test.

** p<0.01.

a ND not detectable.

### Whole Body Ketone Utilization

**Figure 6:**
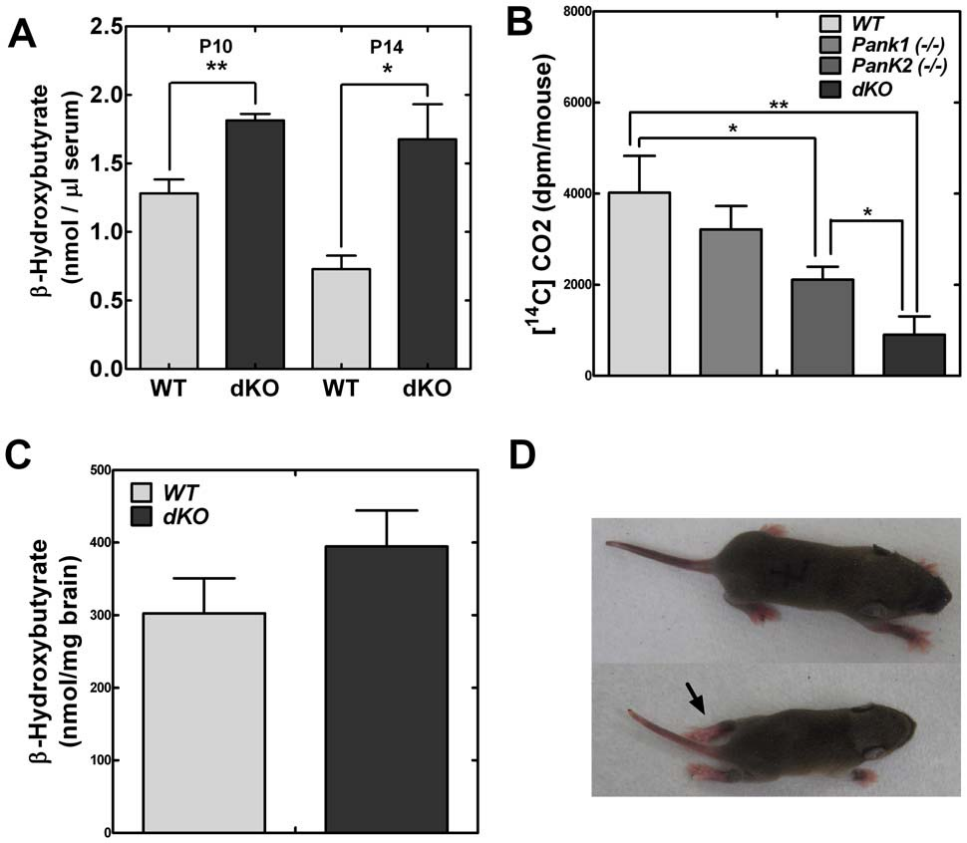
Brain ketone uptake and metabolism. (**A**) β-hydroxybutyrate serum levels in wild type (WT, n = 4) and *Pank 1,2* double knockout mice (dKO, n = 4) at P10, P14. (**B**) [^14^C]β-hydroxybutyrate conversion to [^14^C]carbon dioxide (CO2) in wild type (WT, n = 3), Pank1(−/−) (n = 3), Pank2(−/−) (n = 3), and Pank1,2 double knockout (dKO, n = 3) mice. (**C**) [^14^C]β-hydroxybutyrate uptake by brain from wild type (WT) and double knockout (dKO) mice at P10. (**D**) Dragging of hind limbs (arrow) in double knockout (lower panel) compared to *Pank1(−/−)* littermate (upper panel) observed at P7.

The liver produces both glucose and ketones to supply fuel to the other organs. The brain is a major ketone consumer and utilizes ketones for energy and lipid metabolism when glucose becomes less available. Ketone utilization requires CoA. Previously, the adult *Pank1*(−/−) mice exhibited normal blood ketone levels in the fasted state when glucose levels were depressed [9]. β-Hydroxybutyrate is the major ketone supplied to the circulation by the liver. The serum levels of this ketone were measured in wild-type and dKO mice at P10 and P14 (Fig. 6A). Serum ketone levels were significantly elevated in the dKO mice at P10, and were 60% higher than in wild-type pups by P14. The elevated ketone levels suggested adequate production by the liver, but decreased ketone utilization in the dKO animals. Ketone levels in the dKO brains were slightly but not significantly higher compared to wild-type tissue (Fig. 6C), indicating that ketone uptake was not impaired in the dKO mice. The ability to metabolize ketones was measured by the release of carbon dioxide (CO_2_), a ketone degradation product. Pups were injected with radiolabeled β-[^14^C]hydroxybutyrate in a closed chamber with regulated positive airflow, and after 30 minutes the exhaled radioactive CO_2_ was collected on a filter. The radioactivity on the filter was quantified by scintillation counting. The *Pank2*(−/−) pups produced significantly less [^14^C]CO_2_ compared to wild-type and *Pank1*(−/−) pups, and the dKO mice released less than 75% of the normal wild-type amounts (Fig. 6B). These data indicate that the dKO mice could not efficiently use ketones in the absence of glucose, thus correlating with the elevated ketone levels in the serum.

### Additional *Pank* Knockout Phenotypes

The dKO mice dragged their hind limbs for extended periods between the first and second weeks of the postnatal period. Wild-type mice, *Pank1*(−/−) and *Pank2*(−/−) littermates did not exhibit this characteristic. Both hind limbs were extended outward, and the animals moved forward with their forelimbs (Fig. 6D). The dragging continued in each dKO animal for two to three days, but then was resolved prior to the morbidity that preceeded death. The resolution of the phenotype suggested that the dragging could represent an episode of dystonia in the hind limbs, but the underlying cause is unkown at this time. To the best of our knowledge, the movement disorder occurred in all of the dKO pups and was similar to that described for wild-type mice on a pantothenate-deficient diet [17]. This observation suggests that an overall CoA deficit was responsible, rather than an unrelated but unkown specific function of PanK1 or PanK2. Neither *Pank1(−/−)*, *Pank2(−/−)* nor wild-type matched control animals exhibited this condition during the early postnatal period or later.

Two additional *Pank* dKO strains were derived, using the same methodology described for generation of the *Pank1*/*Pank2* dKO. The *Pank1*/*Pank3* dKO strain was found to be an embryonic lethal combination (0/103 total pups) and the *Pank2*/*Pank3* dKO strain was also embryonic lethal (0/65 total pups). *Pank2*(−/−) mice obtained by gene deletion (this study) were backcrossed onto the C57BL6 background, which was confirmed by genome scanning analysis (Jackson Laboratories). Following the backcross, no viable *Pank2*(−/−) pups were obtained (0/40, seven litters between heterozygous breeders), indicating that the strain background possibly had a modifying effect on the phenotype.

### CoA Levels are Linked to NADH Levels

**Figure 7:**
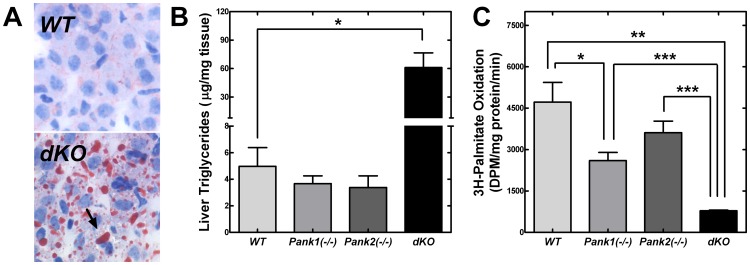
Liver triglyceride levels and fatty acid oxidation. (**A**) Oil Red O staining (arrow) of slices from wild type (WT) and dKO mice. (**B**) Triglyceride levels from P10 wild-type (WT, n = 3), *Pank1(−/−)* (n = 4), *Pank2(−/−)* (n = 6), and dKO, (n = 3) mice. (**C**) Fatty acid oxidation per 30 min per microgram of protein were measured in liver extracts obtained from P10 wild-type (WT, n = 6), *Pank1(−/−)* (n = 4), *Pank2(−/−)* (n = 5), and dKO, (n = 4) mice. * p<0.05; ** p<0.01; *** p<0.001.

Histological examination of the *Pank1/Pank2* dKO P10 pups suggested abnormal accumulation of lipid in the liver. This observation was confirmed using the Oil Red O staining of liver sections which stains neutral lipid droplets (Fig. 7A). In contrast, the livers *Pank1*(−/−), *Pank2*(−/−) and wild-type pups of the same age did not accumulate lipid droplets. *Pank1*(−/−) adult mice accumulate lipid in the liver only after an extended fast and the lipid accumulation correlates with a reduced rate of fatty acid oxidation [9]. Measurements of the neutral lipids in the dKO pups indicated that triglyceride was notably higher in the dKO livers (Fig. 7B), whereas cholesterol and cholesterol esters were similar to those amounts in wild-type livers (not shown). The relative content of the major lipid classes was the same per mg liver weight from wild-type and dKO pups (not shown), with the exception of triglyceride, but the liver weights of the dKO mice were disproportionately smaller (Table 1). These data suggest that bulk synthesis of membrane phospholipid and cholesterol was reduced in the dKO animals, contributing to a developmental delay in liver growth. Measurement of the hepatic CoA-dependent fatty acid oxidation rates in wild-type, *Pank1*(−/−), *Pank2*(−/−) and dKO P10 pups revealed about a 75% reduction in the *Pank1/Pank2*-deficient animals (Fig. 7C). The *Pank1*(−/−) pups exhibited about a 40% reduction, similar to the adult *Pank1*(−/−) mice [9], and the rates of oxidation in *Pank2*(−/−) pups were lower but not significantly different from wild-type pups. Thus, the triglyceride accumulation was attributed to reduced fatty acid oxidation rather than increased lipogenesis in the dKO mice. In support of this concept, the expression levels of PPARα as well as PPARα-target genes were elevated in the *Pank1/Pank2* dKO mice compared to wild-type pups (Table 4). Activation of PPARα promotes expression of genes involved in fatty acid oxidation. Evaluation of expression levels at P10 revealed significant elevation of PPARα, glycerol-3-phosphate acyltransferase 1 (*Gpam*), and acyl-CoA oxidase (*Acox*) in the dKO animals. Expression of genes associated with gluconeogenesis, such as PPARγ-coactivator (*Pgc1α*), phosphoenoylpyruvate carboxykinase (*Pepck1*) and glucose-6-phosphatase (*G6pc*), were unchanged.

**Table 4:** mRNA abundance of genes involved in fatty acid metabolism and gluconeogenesis.

Gene	Wild Type	dKO
*Ppar-α*	0.012±0.006 (6)	0.180±0.060 (6)*
*Gpam*	0.021±0.005 (7)	0.036±0.012 (7)*
*Acsl1*	0.391±0.031 (6)	0.583±0.241 (6)
*Acox*	0.082±0.028 (7)	0.164±0.033 (7)***
*Pgc1α*	0.030±0.015 (3)	0.014±0.002 (3)
*Pepck1*	0.768±0.249 (7)	0.866±0.154(7)
*G6pc*	0.343±0.118 (7)	0.337±0.154 (7)

Transcript abundance was measured relative to *Gapdh* (Glyceraldehyde 3-phosphate dehydrogenase) as a calibrator. Transcripts included peroxisome proliferator-activated receptor alpha (*Ppar-α*), glycerol-3-phosphate acyltransferase 1 (*Gpam*), long-chain fatty acyl-CoA ligase 1 (*Acsl1*), acyl-CoA oxidase (*Acox*), peroxisome proliferator-activated receptor gamma coactivator 1-alpha *(Pgc1-α),* phosphoenoylpyruvate carboxykinase (*Pepck*) and glucose-6-phosphatase (*G6pc*). Transcripts were measured in triplicate and combined results are shown ± s.d. (number of mice). Significance calculated using Students t-test.

* p<0.05;

** p<0.01;

*** p<0.001.

**Figure 8:**
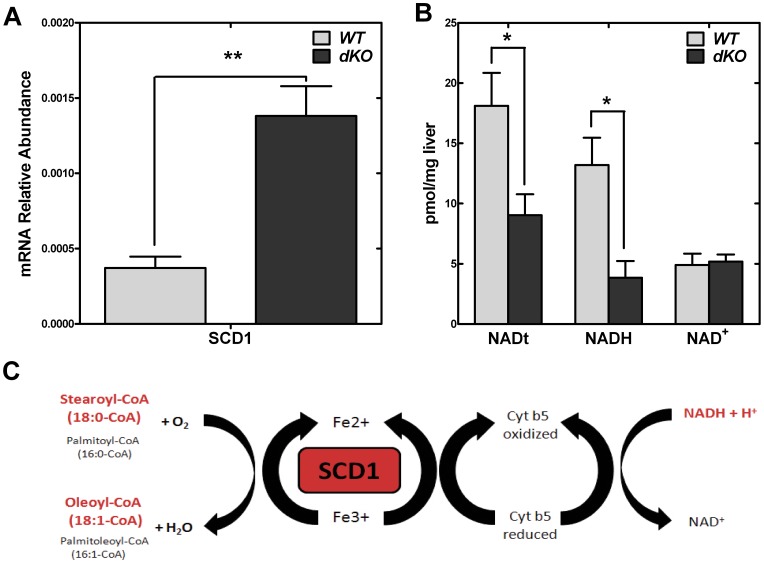
Steroyl-CoA desaturase expression and NAD levels. (**A**) Transcript abundance for stearoyl CoA dehydrogenase (*Scd1*) from P10 wild type (WT, n = 4) and dKO (n = 4) relative to transcript abundance of glyceraldehyde 3-phosphate dehydrogenase. Each transcript was measured in triplicate. (**B**) Levels of total nicotinamide adenine dinucleotide (NADt), reduced nicotinamide adenine dinucleotide (NADH) and oxidized nicotinamide (NAD^+^) from P10 wild type (WT, n = 4) and dKO (n = 4) mice. (**C**) Scheme of conversion of stearoyl-CoA (18∶0) to oleoyl-CoA (18∶1) mediated by stearoyl CoA desaturase (SCD1). * p<0.05; ** p<0.01 where indicated.

Analysis of the liver fatty acids showed that the composition was the same in livers from wild-type and dKO pups, with the exception of the C16 and C18 chain lengths (Table 5). The relative amount of C18∶0 was greater and the amounts of C16∶1 and C18∶1 were significantly lower in the dKO animals, suggesting a deficiency in stearoyl-CoA desaturase (SCD) activity. To explore this possibility, the levels of *Scd1* transcripts (Fig. 8A) in livers from wild-type and dKO P10 pups were quantified. Surprisingly, SCD1 expression was more than 3-fold higher in the dKO livers. As the supply of C16∶0 and C18∶0 substrates were the same or higher than those encountered in wild-type livers, we investigated whether the amount of NADH was reduced and perhaps limiting for SCD activity. NADH is a required cofactor for SCD1 activity (Fig. 8C) and is produced by glycolysis and/or CoA-dependent fatty acid oxidation. The amount of NADH was >50% lower in dKO pups compared to wild-type (Fig. 8B), shifting the NAD^+^/NADH ratio from 0.4 to 1.3. These data indicated that the supply of NADH was possibly limiting for synthesis of C18∶1 and suggested that the increased expression of *Scd1* was an adaptive response to promote C18∶1 production in the dKO livers. Altogether, these results indicated that fatty acid metabolism was impaired due to restriction of the CoA levels in the *Pank1/Pank2* dKO mice, although the gene expression changes that promote fatty acid metabolism and gluconeogenesis were intact. The reduced NADH levels in the dKO mice, due at least in part to a substantially reduced rate of CoA-dependent fatty acid oxidation, likely contributed to the deficit in SCD activity and the impairment of gluconeogenesis, as both processes are strictly dependent upon the availability of this cofactor.

**Table 5:** Fatty acid profile of livers from wild type and dKO mice.

Fatty Acid^a^	Wild Type (%)	dKO (%)
Myristic Acid (14∶0)	2.55±0.40 (3)	1.51±0.33 (3)
Palmitic Acid (16∶0)	27.43±1.20 (3)	32.30±2.77 (3)
Palmitoleic Acid (16∶1)	1.50±0.26 (3)	0.23±0.23 (3)*
Stearic Acid (18∶0)	12.17±2.19 (3)	22.71±2.43 (3)*
Oleic Acid (18∶1)	18.11±2.68 (3)	9.125±0.46 (3)*
α-Linoleic Acid (18∶2)	19.27±1.17 (3)	13.04±1.55 (3)*
Arachidonic Acid (20∶4)	9.06±1.10 (3)	11.10±2.05 (3)
Docosahexaenoic Acid (22∶6)	7.84±1.20 (3)	7.35±2.45 (3)

a Fatty acid abundance ≥1%.

Significance calculated using Students t-test.

* p<0.05.

## Discussion

The relatively mild phenotypes in each of the single PanK knockout mice show that each PanK isoform can be mostly compensated for by the expression of the other two isoforms in most tissues. The postnatal lethality of the *Pank1/Pank2* dKO mice coupled with the embryonic lethality of *Pank1/Pank3* and *Pank2/Pank3* dKO mice illustrate that normal development requires at least two functional PanK isoforms. The contribution of each PanK to tissue CoA levels is related to their relative expression levels. PanK1 activity is more important in maintaining CoA levels in liver where it is most highly expressed [9], whereas PanK2 is more important in brain where it is the predominant activity (Fig. 5). The tolerable reduction in fatty acid oxidation and gluconeogenesis evident in the *Pank1* KO mouse [9] is exacerbated in the *Pank1/Pank2* dKO mouse. The *Pank1/Pank2* dKO animals are severely hypoglycemic, and their livers are defective in fatty acid oxidation as reflected by the accumulation of triglycerides (Fig. 7). The *Pank1/Pank2* dKO mouse also develops a marked hyperketonemia by P10 and fails to oxidize ketones (Fig. 6). This phenotype points out the critical importance of PanK regulation of CoA levels in cellular activation and energy production from ketones. Ketone bodies provide fuel to brain, heart, and skeletal muscle in metabolic states that include starvation, low carbohydrate diets, and during neonatal development. The high fat milk diet provided to suckling pups provides fatty acids for oxidative metabolism in cardiomyocytes and skeletal myocytes [22], but most neurons oxidize fatty acids poorly and therefore depend on hepatic gluconeogenesis and ketogenesis for energy [23], [24], [25]. The critical role for ketone utilization in neonatal metabolism is revealed using mice lacking the ketolytic enzyme succinyl-CoA:3-oxo-acid CoA-transferase encoded by *Oxct1* [26]. These knockout mice die within 48 hours of birth. The *Pank1/Pank2* dKO phenotype is slightly less severe because it deprives the enzymes of ketone metabolism of their required cofactor, but the enzymes are not completely inactivated as in the *Oxct1* knockout mouse.

This study highlights the important relationship between PanK-dependent regulation of the cellular CoA concentration and the maintenance of NADH levels. The marked deficiency in the hepatic capacity for β-oxidation in *Pank1/Pank2* dKO mice appears directly linked to the severely depressed level of NADH that translates into a two-fold increase in the NAD^+^/NADH ratio. This impaired generation of reducing power due to CoA depletion was reflected by the deficit in oleic acid, the major monounsaturated fatty acid produced by liver. This occurs in spite of a significant upregulation in the expression of the key enzyme in the pathway, NADH-dependent SCD-1. The inability to produce a sufficient supply of NAD(P)H also directly impacts gluconeogenesis, which involves several reductive steps. The NAD^+^/NADH ratio is often used as an indicator of the metabolic and redox state of the cell, and this parameter regulates the activity of key metabolic enzymes such as pyruvate dehydrogenase [27]. Additionally, the NAD^+^/NADH ratio modulates the activity of the sirtuin Sirt1 [28], a NAD^+^-dependent deacetylase that plays a central role in the posttranslational regulation of metabolic enzymes and numerous transcription factors by removing acetyl groups at specific lysine residues [29], [30], [31]. Reversible protein acetylation has recently emerged as a major, conserved poststranslational modification of thousands of non-histone proteins in the cytosol and mitochondria [32]. The idea that acetyl-CoA levels or the acetyl-CoA/CoA ratio is equally important in influencing the extent of protein acetylation is supported by the observation that a reduction in CoA levels due to PanK deficiency reduces histone and tubulin acetylation in *Drosophila* [33]. Thus, the reduced hepatic NADH coupled with lower acetyl-CoA levels both contribute to the severity of the phenotype arising in *Pank1*/*Pank2* dKO mice by directly and indirectly altering the activity of key metabolic enzymes and global transcription [34], [35].

The severity and complexity of the *Pank1/Pank2* dKO phenotype precludes its utility in the study of the pathobiology of PKAN disease. PKAN disease arises from mutations in the human PanK2, most of which inactivate PanK2. This leads to the idea that the inability of human brain to maintain cellular CoA underlies the neurodegeneration that is the hallmark of PKAN. Human PanK2 has the same biochemical regulation as the murine PanK2, but the human isoform localizes to mitochondria whereas the murine isoform is found primarily in the cytosol [11]. An earlier report from Johnson et al. [36] indicated that the mouse PanK2 was predominantly associated with mitochondria. However, all of the cell lines used to make this conclusion were derived from humans or primates, not mice. Inspection of the immunocytochemistry data in the report by Johnson et al., reveals that localization of the endogenous antigen recognized by the mouse anti-PanK2 antibody was found throughout the primate cell, and mitochondrial staining constituted only a small fraction of the total. Finally, the cellular images in the report by Johnson et al., were obtained using a conventional microscope, not a confocal microscope, thereby increasing the likelihood of fortuitous co-localization due to coincidence of the antigen staining and the mitochondrial staining in distinct focal planes. *Pank2*(−/−) mice do not exhibit any symptoms of PKAN disease [12]. It is not clear whether the absence of a mouse brain phenotype is due to the different localization of PanK2, the higher expression levels of the other isoforms relative to *Pank2* in mouse brain compared to human [11], or if other factors account for the differences between mouse and human physiology. The low expression of *Nudt19* in the whole brain (Table 3) does not address expression in different regions of the central nervous system which may differ in abundance or functionality between postnatal and adult mice (http://203.181.243.69/brain/Nudt19/69609073/thumbnails.html; http://www.ebi.ac.uk/gxa/gene/NM_001004258http://mousespinal.brain-map.org/imageseries/show.html?id=100020947). *Nudt19* is more highly expressed in the adult hippocampus, cerebellum, and in the cell bodies of the nerves in the dorsal and ventral horns, compared to other specific regions of the brain and spinal cord. The distribution of *Nudt19* in regions of the postnatal brain as it contintues to develop are not known, although it’s expression can be used as an early biomarker in a mouse model of Alzhemiers disease [37]. One reason for our development of the series of *Pank* dKO mice was to find a combination that would lower brain CoA levels and induce a neurodegenerative phenotype. Although the Pank1/Pank2 dKO mice have lower brain CoA, the severity of the global metabolic phenotype precludes using this model to study PKAN. Our experiments suggest that the combination of a *Pank1*(−/−) global knockout coupled with a brain specific deletion in *Pank2* may be a viable approach to generating a model for PKAN disease.

## Materials and Methods

### Ethics Statement

All procedures were performed according to protocol 323 which was approved by the St. Jude Children’s Research Hospital Institutional Animal Care and Use Committee.

### Generation of *Pank2*(−/−) and *Pank1*(−/−)/*Pank2*(−/−) Mice

The *Pank2* targeting construct (pPJ259) was generated by cloning 3 fragments of mouse *Pank2* genomic DNA into vector (pNEOtkLoxP). Plasmid pPJ259 contained a loxP site between a 3.53-kb genomic DNA fragment and the TK and NEO selection cassettes, followed by a second loxP site upstream of a 2.78-kb genomic DNA fragment containing exon 3, and by a third one upstream of 1.81-kb genomic DNA (Fig. 1A). The targeting vector was digested with DraIII restriction enzyme prior to transfection into the mouse embryonic stem (ES) cell line derived from strain 129/SvEv (Specialty Media), grown on mitotically inactivated mouse embryonic fibroblasts which were resistant to neomycin. Clones resistant to the neomycin analog G418 were selected and screened by Southern blot analysis. Genomic DNA from individual ES cell clones was digested with BamHI and separated on a 0.7% agarose gel. DNA was transferred to nylon membrane (Hybond™-N^+^, Amersham Pharmacia Biotech) and hybridization was performed using HybrisolI solution (Intergen). A 772-bp XbaI digested probe fragment containing exon 5 and downstream from the targeted region was labeled with ^32^P-dCTP using the RediprimeII DNA Labeling System (Amersham) and hybridized to the blot at 42 degrees C overnight. After washing, the blot was exposed to a phosphor screen for 1–3 days and scanned. The presence of a 10.4-kb band indicated ES cells that had undergone homologous recombination as opposed to the 12.8-kb wild-type band. The presence of the third loxP site was confirmed by performing PCR with primers LoxPfor (5′-ACTGTAACAAGATTGCCTTGAG) and LoxPrev (5′-AGACATCATGAACAAGACAACC) and then digesting the PCR product with ClaI. The wild type allele yielded a product of 480 bp; the allele with the loxP site yielded two bands of 231 and 229 bp. Embryonic stem cells containing the recombined *Pank2* DNA at the correct locus were injected into C57BL/6J (Jackson Laboratories) mouse blastocysts, which were then implanted into pseudopregnant female mice by the St. Jude Transgenic Core Facility. Male chimeric offspring with 75% to 90% agouti color, the coat color contributed by the embryonic stem cells, were bred with C57BL/6J females. Pups that were 100% agouti, indicating germ line transmission, were screened. Tail clips were lysed and multiplex PCR analysis was used to genotype the mice using RED Extract-N-Amp PCR kit (Sigma) and three primers: F (5′-TTCCCTGCTTAGGTAGGATTGC), R1 (5′-CCTTTGGACACCATGTAAATGAAC) and T (5′-CCAAGTTCGGGTGAAGGC). The wild type allele yielded a product of 618-bp, and the recombinant floxed *Pank2* allele yielded a product of 452-bp. FVB/N-Tg(ACTB-cre)2 Mrt/J mice (Jackson Laboratories) expressing Cre recombinase driven by the human β-actin gene promoter were utilized to generate a deletion within the floxed *Pank2* gene by recombination of the first and third loxP sites, resulting in the loss of exon 3. A multiplex PCR analysis was used to genotype the offspring from the breeding of ACTB-cre mice with mice heterozygous for the floxed *Pank2* using primers F, R1, and R2 (5′-CCTCAACTCCTAGATCCAAACTG). A 618-bp product indicated the presence of a wild type allele, and a 526-bp product indicated the presence of a knockout allele. Generation of the *Pank1(−/−)* was previously described [9]. Initial generation of the *Pank1(−/−)/Pank2(−/−)* double knockout (dKO) mice was done by breeding strains *Pank1 (+/−)* with *Pank2(+/−).* Subsequent litters of dKO mice were derived by mating *Pank1(+/−) and Pank2(−/−)* littermates. Subsequent litters of wild type control mice were derived by mating the wild type littermates from the initial breeding pairs.

### Animal Studies

Mice were maintained at a room temperature of 72°±2°F, room humidity of 50%±10%, and a 14-hr light, 10-hr dark cycle, with the dark cycle starting at 20∶00 hr. Water and chow (Lab Diet 5013) were supplied ad libitum. Blood glucose levels were measured via tail bleeding using a glucometer (FreeStyle). Tissue was dissected from the animal and incubated at 4 degrees C overnight in 10% formalin prior to dehydration in ethanol and embedding in paraffin. Paraffin sections were cut to 4 µm thickness. Sections were stained with hematoxylin and eosin. Sample preparation was performed in the Animal Diagnostic Lab and slides were viewed with an Olympus BX41 microscope at low and high magnification. Evaluation of brain included cerebral cortex, basal ganglia, diencephalon midbrain, brain stem and cerebellum. Evaluation of heart included atrioventribular valves and the cardiac muscle associated with the free wall of the right and left ventricle, the interventribular septum and the right and left atrium.

### Measurements of Gene Expression, PanK Activity, and Tissue CoA Levels

For RNA isolation, mice were euthanized and the tissues were quickly excised and immersed in RNAlater (Ambion). RNA was extracted using Trizol reagent according to the manufacturer’s instructions (Invitrogen). Genomic DNA was removed by digestion with turbo DNASE free (Ambion) and cDNA was synthesized by reverse transcription using SuperScript™ II RNase H^−^ reverse transcriptase, random primers and the RNA templates. Quantitative real-time PCR was performed in triplicate with the primers and listed in Table 6 and SYBR Green. The Taqman rodent GAPDH (Applied Biosystems) was used as a control. All of the values were compared using the C_T_ method [38], and the amount of cDNA (2^−ΔCT^) was reported relative to glyceraldehyde-3-phosphate dehydrogenase (GAPDH) mRNA. For measurements of tissue PanK activity or CoA, tissues were flash frozen in liquid nitrogen. To determine the PanK activity, 200 mg of frozen tissue was homogenized in cold buffer (1 ml, 20 mM K_2_HPO_4_, 1 mM ATP, pH 7.4) using a Dounce tissue grinder. The homogenates were centrifuged at 20,000×g, 4 degrees C for 45 min, and the supernatants were dialyzed overnight in the homogenization buffer to remove small metabolites such as acetyl-CoA. Protein was determined by the Bradford method [39]. The dialyzed samples were diluted to 20 mg protein/ml in 20 mM K_2_HPO_4_, pH 7.4, and the PanK activity was assayed in reaction mixtures containing 100 mM Tris-HCl, pH 7.4, 10 mM MgCl_2_, 2.5 mM ATP, 90 µM D-[1-^14^C]pantothenate (specific activity, 27.5 mCi/mmol, American Radiolabeled Chemicals), 0–200 µg of homogenate protein. Samples were incubated at 37 degrees C for 30 min, after which time the reactions were stopped with the addition of 4 µl of 10% acetic acid into the 40 µl samples, and then analyzed as previously described [2]. CoASH and CoA thioester levels were determined as previously described [8].

**Table 6:** List of primers used for quantitative real time PCR.

Gene	Forward Primer (5′→3′)	Reverse Primer (3′→5′)
**Pank1**	ATGACTTGCCCTCATTTGCAT	TGGGAGCCCCTCCAAATT
**Pank2**	TTGGGCATACGTGGAGCTTT	TCTCACATACATTTCAACAGGACAAG
**Pank3**	CTAAGGAACGCCTGCCATTC	CTTTGGTTCCCAGTGACAGACA
**Nudt7**	CCAAGTGGAGGTGGTCTCTC	GATGAAATCACGGCCAGACT
**Nudt19**	ATCTGTGCCATCCGCGAAGC	CACAGCTGGAGGAAGCAGCG
**Ppar-α**	TGGCAAAGGCAAGGAGAAG	CCCTCTACATAGAACTGCAAGGTTT
**Pepck1**	GCTGGCAGCATGGGGTGTTT	TTGGGCAACTTGGCTGCTGG
**G6pc**	CTTTCAGCCACATCCGGGGC	CCCATTCTGGCCGCTCACAC
**Acox**	CCAGGCCACCGAAGAGCAGC	CCAGGCCACCGAAGAGCAGC
**Acsl1**	CTACCGTCCTGGGCACAGA	TGTTCCTTGCACAGTTCTTCGA
**Gpam**	GTCATACCCGTGGGCATCTC	TCCCAACTGTTCGCCATTGT
**Pdh1**	CCGCTATGGCATGGGGACGT	GCTCCATCAGGATGGGCCCCTTAC
**Aaca1a**	CCCTTTCAGGCCTTGCGACGT	ATCGCGGAGGTGGGTCCTACC
**Hmgcl1**	CCTCCGGAGTGAAGATGGCGTCAG	GTAGGGGCATCCGAGGGCACA
**Oxct1**	CTGCATAAGGGGTGTGTCTGCTACT	CTCCACCGGCACGGATCCTCT
**Bdh1**	CCTGCCGGCTTGCTTTGGGAG	TTCAGGCCGGAGCGGATCGT
**Scd1**	ATCACCGCGCCCACCACAAG	AACTCAGAAGCCCAAAGCTCAGCT

### Lipid Analysis

Lipids were extracted from 50 mg of tissue using a modification of the Bligh and Dyer procedure optimized for lipid quantitation by the LipidMaps group [40]. The amount of each major lipid class was determined by flame-ionization detection following thin layer chromatography using an Iatroscan MK-5 (Iatron, Tokyo, Japan). Triglycerides, cholesterol and cholesterol esters were separated using hexane:ether (90/10, v/v). The lipids were identified by co-migration with authentic standards and quantified by comparison with known amounts of pure standard lipids (Avanti Polar Lipids). The amounts of each fatty acid, including free fatty acid and lipid fatty acyl groups, were determined from 50 mg of tissue. The tissue was extracted as described above [40] and methyl esters were prepared followed by hexane extraction. The amount of each fatty acid methyl ester was determined by flame-ionization detection following gas chromatography using a Hewlett Packard 5890A. The fatty acids were identified by co-migration with authentic standards and quantified by integration of the signal peaks.

### Measurement of NAD/NADH

Total NAD [NAD^+^ plus NADH] and NADH levels were determined according to instruction provided with the NAD/NADH colorimetric assay kit from Abcam. Approximately 20 mg of tissue was homogenized in 400 µl of extraction buffer and was deproteinized using perchloric acid (Abcam) prior to the determination.

### Histology

Hematoxylin and eosin (H&E) and Oil Red O (ORO) staining of liver sections was performed by the Veterinary Pathology Core at St. Jude Children’s Research Hospital. For H&E staining, the livers were fixed in 10% formalin. ORO staining was performed on frozen sections embedded in Shandon Cryomatrix (Thermo Scientific). The sections were imaged with an Olympus BX41 Microscope equipped with a Diagnostic Instruments RTKE camera.

### Measurement of Fatty Acid Oxidation

The procedure described by Adams *et al.* [41] was adapted to measure the rate of [9,10-^3^H]palmitic acid oxidation. Briefly, tissues were excised, rinsed in cold isolation buffer (220 mM mannitol, 70 mM sucrose, 2 mM Hepes, 0.1 mM EDTA, pH 7.2), blotted dry, pooled and weighed. The tissues were homogenized in isolation buffer to a final concentration of 400 mg of tissue/ml. The reaction buffer contained 1 mM L-carnitine, 13.1 mM sucrose, 78 mM Tris-HCl, 10.5 mM K_2_HPO_4_, 31.5 mM KCl, 5 mM ATP, 1 mM NAD+, 850 µM EDTA, 500 µM palmitic acid, 1.7 µM of [9,10-^3^H]palmitic acid (60 Ci/mmol; American Radiolabeled Chemicals) and 100 mg/ml fatty acid-free BSA, pH 7.4. Ethanol stock solutions of radiolabeled and unlabeled palmitic acid were added directly to the reaction buffer. Reactions were started by the addition of 100 µl of tissue homogenate to 250 µl of reaction buffer and incubated at 37°C for 30 min. The assay was confirmed to be linear for 30 min by prior time course experiments. At these time points the reactions were stopped by adding 150 µl of 30% perchloric acid and centrifuged at 20,000×g, 4 degrees C for 2 min to pellet the precipitated proteins. Pellets were washed with 0.5 ml water and centrifuged again. The two supernatants were combined and the [^3^H] water produced by β-oxidation in each sample was separated from [9,10-^3^H]palmitic acid using the lipid extraction procedure described by Bligh and Dyer [42]. Aliquots of the aqueous phases were transferred to scintillation vials and radioactivity was quantified.

### Measurement of Ketone Utilization

Serum was obtained from mice at P10 and P14. The serum concentration of β-hydroxybutyrate was measured using the Biovision colormetric assay kit according to the manufacturer’s directions. To measure β-hydroxybutyrate tissue uptake and utilization, 3 µCi of β[1-^14^C]hydroxybutyric acid (American Radiolabeled Chemicals, specific acitivity 50–60 mCi/mmol) in phosphate-buffered saline, pH 7.2 (final volume 100 µl) was supplied by intraperitoneal injection to mice at P10. Immediately thereafter, mice were placed in an airtight chamber with continuous forced airflow for 30 minutes and the released ^14^C-carbon dioxide was captured on Millipore glass fiber filters (Millipore; Cat. No. AP4001000) pre-soaked in 1 M NaOH. Dry disks were placed in Hi-Ionic Scintillation Fluid (Perkin-Elmer) and radioactivity was determined by scintillation counting. Thereafter, mice were euthanized, tissues were excised, weighed and the amount of radioactive β-hydroxybutyric acid was determined following solubilization in 10 µl per mg of tissue of Solvable (Perkins-Elmer) and incubation at 55 degrees C for 60 min. Coloration of the samples was cleared by incubation in 30% hydrogen peroxide. Then, samples were centrifuged at 2655×g for 5 minutes to pellet insoluble debris and 100 µl of supernatant was added to 3 ml of scintillation fluid, mixed, and radioactivity was determined.
